# Stochastic and Deterministic Effects of a Moisture Gradient on Soil Microbial Communities in the McMurdo Dry Valleys of Antarctica

**DOI:** 10.3389/fmicb.2018.02619

**Published:** 2018-11-01

**Authors:** Kevin C. Lee, Tancredi Caruso, Stephen D.J. Archer, Len N. Gillman, Maggie C.Y. Lau, S. Craig Cary, Charles K. Lee, Stephen B. Pointing

**Affiliations:** ^1^Institute for Applied Ecology New Zealand, Auckland University of Technology, Auckland, New Zealand; ^2^School of Biological Sciences, Queen’s University Belfast, Belfast, United Kingdom; ^3^Institute for Global Food Security, Queen’s University Belfast, Belfast, United Kingdom; ^4^Department of Geosciences, Princeton University, Princeton, NJ, United States; ^5^International Centre for Terrestrial Antarctic Research, University of Waikato, Hamilton, New Zealand; ^6^Yale-NUS College, National University of Singapore, Singapore, Singapore; ^7^Department of Biological Sciences, National University of Singapore, Singapore, Singapore

**Keywords:** Antarctica, dry valleys, hyporheic, oligotrophic, soil bacteria, soil fungi, water availability

## Abstract

Antarctic soil supports surface microbial communities that are dependent on ephemeral moisture. Understanding the response to availability of this resource is essential to predicting how the system will respond to climate change. The McMurdo Dry Valleys are the largest ice-free soil region in Antarctica. They are a hyper-arid polar desert with extremely limited moisture availability. Microbial colonization dominates this ecosystem but surprisingly little is known about how communities respond to changing moisture regimes. We utilized the natural model system provided by transiently wetted soil at lake margins in the Dry Valleys to interrogate microbial responses along a well-defined contiguous moisture gradient and disentangle responses between and within phyla. We identified a striking non-linear response among bacteria where at low moisture levels small changes resulted in a large impact on diversity. At higher moister levels community responses were less pronounced, resulting in diversity asymptotes. We postulate that whilst the main drivers of observed community diversity were deterministic, a switch in the major influence occurred from abiotic factors at low moisture levels to biotic interactions at higher moisture. Response between and within phyla was markedly different, highlighting the importance of taxonomic resolution in community analysis. Furthermore, we resolved apparent stochasticity at high taxonomic ranks as the result of deterministic interactions taking place at finer taxonomic and spatial scales. Overall the findings provide new insight on the response to moisture and this will be useful in advancing understanding of potential ecosystem responses in the threatened McMurdo Dry Valleys system.

## Introduction

The McMurdo Dry Valleys of Antarctica constitute the largest ice-free region on the continent (SCAR, 2004). This extreme polar desert presents exceptional environmental and resource challenges for life (Convey et al., 2014). Water bio-availability is limited to a short period during the austral summer when temperatures allow ice and snow to melt (Pointing et al., 2015). Microhabitats within and beneath rocks support patchy islands of cryptic but well-developed microbial colonization (Yung et al., 2014; Wei et al., 2016; Archer et al., 2017). Soils, however, are a poorly developed oligotrophic and moisture-limited microbial habitat and consequently they support extremely low standing biomass characterized by heterotrophic bacteria (Niederberger et al., 2008; Pointing et al., 2009; Lee et al., 2012). Terrestrial meltwater ponds and streams occur where liquid water can accumulate throughout the Dry Valleys and these aquatic systems support extensive colonization dominated by cyanobacterial mats (Vincent et al., 1993; Vincent, 2002). The coupling of these meltwater features to water input/output also result in expansive wetted hyporheic soils where water is retained over several weeks during the growing season (Gooseff et al., 2003) and creates favorable environments for microbial colonization (Ball and Levy, 2015). Developing an understanding of microbial response to moisture as the primary driver of habitability is essential to predicting potential impacts on Dry Valleys ecology in a landscape on the threshold of climate-induced change (Fountain et al., 2014).

Evidence for microbial community responses to moisture is scarce and somewhat contradictory for hyporheic soils since most previous studies have focused on the aquatic environment: A study of transiently wetted Antarctic hyporheic soils identified a defined community structure between moisture-sufficient (Cyanobacteria-dominated) and <5% moisture soils (Acidobacteria, Actinobacteria, Deinococci and Bacteroidetes-dominated) (Niederberger et al., 2015b), although another comparing Antarctic and hot desert soils did not (Zeglin et al., 2011). Where low moisture soils were experimentally augmented with water and nutrients a drive toward desiccation tolerant taxa Acidobacteria, Firmicutes and Proteobacteria (Buelow et al., 2016), or Actinobacteria and Bacteroidetes (Tiao et al., 2012) was observed. This highlights the complexity of community response when diverse taxa within and between phyla may be present and respond differently along an environmental gradient.

It is reasonable to assume that deterministic processes drive community assembly in response to moisture for this system since this is the key factor determining habitability in the Dry Valleys (Cowan et al., 2014). The deterministic model predicts niche partitioning will result in segregation in terms of species co-occurrence or even aggregation if niche partitioning interacts with environmental stochasticity (Chave, 2004; Dornelas et al., 2006; Chase, 2007), such as a meltwater moisture regime in the Dry Valleys. The Antarctic system is an ideal model against which to test this due to the extreme oligotrophic nature of soils and lack of trophic complexity. It is also reasonable to assume that bacterial taxa do not display the same response to abiotic variables and this has been suggested by one recent study that revealed striking differences even at a simple delineation between photoautotrophs and heterotrophs (Caruso et al., 2011). We therefore predict considerable heterogeneity in moisture response may occur among diverse taxonomic groups.

Microbial response to moisture availability along a contiguous gradient remains poorly defined for Antarctic Dry Valleys soil. In addition, previous studies employed typical distance-related comparisons of communities and so whilst broad trends in overall community have been observed, the approach may have obscured potential differential responses at finer taxonomic resolution, and the latter is more informative with regard to functionality (Fierer et al., 2007). Here we report bacterial community response to a contiguous and well-defined moisture gradient at an unprecedented level of taxonomic resolution. We identify support for a deterministic process driving bacterial diversity shifts in response to moisture availability and establish for the first time that a striking non-linear response to moisture occurs. The findings provide critical new insight on the response to moisture and will allow better predictions of resilience in the threatened McMurdo Dry Valleys system.

## Materials and Methods

### Field Sampling

Sampling was conducted around the hyporheic zone of Spaulding Pond, Taylor Valley in the McMurdo Dry Valleys of Antarctica, during the austral summer of 2015. Four transects were defined in north-east (S77° 39.489′, E163° 07.501′), south-east (S77° 39.513′, E°163 07.626′), south (S77° 39.589′, E°163 07.006′), and north-west (S77° 39.470′, E°163 06.336′) facing hyporheic moisture gradients. We adopted a zonal sampling approach where soils were retrieved that matched visible delineations along linear transects extending across the hyporheic zone from the water’s edge at each sampling station: Zone (1) Saturated soil adjacent to water’s edge; Zone (2) Wet soil as indicated by dark coloration; Zone (3) Ephemerally wet soil, dry but with evidence for previous water indicated by surface evaporites; Zone (4) Dry soil with no indication of recent moisture. Soils from each zone in each transect were recovered using aseptic technique and saturated with Lifeguard solution (Qiagen, Netherlands) (*n* = 16) with parallel sampling for geochemical and moisture analysis (*n* = 16). All samples were stored in darkness frozen at -20^o^C until processed.

### Soil Analysis

Moisture content was estimated gravimetrically after drying soil samples to constant dry mass at 120^o^C for 48 h. Soil chemical analyses for variables known to affect microbial colonization, including pH, extractable cations, cation exchange capacity, phosphorous and sulfur were measured according to standard chemical analysis methods (Blakemore et al., 1987; Landcare Research^1^). In brief, geochemical tests were conducted using the following methodology: All sediments were dried in a forced air convection drier at 35°C, and after drying, sediments were crushed to pass through a 2 mm sieve. For pH, 10 mL of sediment was slurried with 20 mL of water, and after standing, the pH was measured (1:2 v/v slurry). Cations (K^+^, Ca^2+^, Mg^2+^, Na^+^) were extracted using ammonium acetate (1.0M, pH 7, 1:20 v/v sediment:extractant ratio, 30 min extraction), and determined by ICP-OES. Cation Exchange Capacity (CEC) was calculated by summation of the extractable cations and the extractable acidity. Phosphorus was extracted using Olsen’s procedure (0.5M sodium bicarbonate, pH 8.5, 1:20 v/v sediment:extractant ratio, 30 min extraction), and the extracted phosphate was determined calorimetrically by a molybdenum blue procedure. For sulfate-sulfur, sediments were extracted using 0.02M potassium dihydrogen phosphate after 30 min shaking, and sulfate-sulfur was measured by anion-exchange chromatography (IC). Total nitrogen (TN) and total carbon (TC) were determined by the Dumas method of combustion. Each sample was combusted to produce varying proportions of CH_4_ and CO gas. The CH_4_ and CO gas was oxidized to CO_2_ using the catalysts Copper Oxide and Platinum. The CO_2_ was measured using Thermal Conductivity detector. Available nitrogen was estimated after incubating sediment samples for 7 days at 40°C, after which the ammonium-N was extracted with potassium chloride (2M potassium chloride, 1:5 v/v sediment:extractant ratio, 15 min shaking), and determined calorimetrically. Moisture was measured in percentage w/w; pH in pH unit; Olsen P (bicarbonate-extractable P), K, Ca, Mg, Na, and S (Sulfur and Sulfate) were measured in mg per kg of soil; cation-exchange capacity (CEC) was measured in me per 100 g of soil.

### Environmental 16S rRNA Gene-Defined Diversity

Samples were thawed on ice and three 0.5 g extractions were conducted using the CTAB method optimized for Antarctic oligotrophic environmental samples (Archer et al., 2015). DNA yield was measured in ng/g soil. Illumina MiSeq libraries were prepared as per manufacturer’s protocol (Metagenomic Sequencing Library Preparation Part # 15044223 Rev. B; Illumina, San Diego, CA, United States) as previously described (Lee et al., 2016). PCR targeting the V3–V4 regions of bacterial and archaeal 16S rRNA gene with the primer set: PCR1 forward (5′ TCGTCGGCAGCGTCAGATGT GTATAAGAGA CAGCCTACGG GNGGCWGCAG 3′) and PCR1 reverse (5′GTCTCGTGGG CTCGGAGATG TGTATAAGAG ACAGGACTAC HVGGGTATCT AATCC 3′) was conducted using KAPA HiFi Hotstart Readymix (Kapa Biosystems, Wilmington, MA, United States) and the following thermocycles: (1) 95°C for 3 min, (2) 25 cycles of 95°C for 30 s, 55°C for 30 s, °C for 30 s, 72°C for 5 min, and (3) holding the samples at 4°C. The amplicons were then indexed using Nextera XT index kit (Illumina). AMPure XP beads (Beckman-Coulter, Brea, CA, United States) was used to purified the amplicon. Sequencing was conducted with an Illumina MiSeq system (Illumina) with the 500 cycle V2chemistry at Auckland University of Technology, New Zealand. A 5% PhiX spike-in was used, as per manufacturer’s recommendation.

### Data Processing

The paired-end reads were merged using USERCH v.9.0.2132 (Edgar, 2013). The merged reads were then filtered to remove extraneous sequences that were unlikely to be the target marker amplicon (16S rRNA gene). Mothur v1.36.1 (Schloss et al., 2009) was used to remove sequences outside of 200–500 bp range or containing > 6 homopolymers. Low quality sequences (>1 expected error) and singletons (except for when calculating bacterial richness and diversity) were removed to reduce false identification of operational taxonomic units (OTUs). These curated high quality sequences were clustered *de novo* with USEARCH using a 97% identity threshold into OTUs. The representative OTU sequences were taxonomically classified using the RDP classifier (Wang et al., 2007) implemented in QIIME v9.1.1 (Caporaso et al., 2010) with Greengenes 13_8 reference database (McDonald et al., 2012). From the 16 samples, a total of 4.3 GB (gzipped) of data was generated. The processing identified 321 OTUs, and resulted in a total of 1,173,355 reads and a mean sampling depth of 73,334 reads. All sequence data acquired during this investigation has been deposited in the EMBL Sequence Read Archive (SRA) as BioProject PRJEB27415 under accession numbers ERS2573055 to ERS2573070.

### Statistical Analyses

**FIGURE 1:**
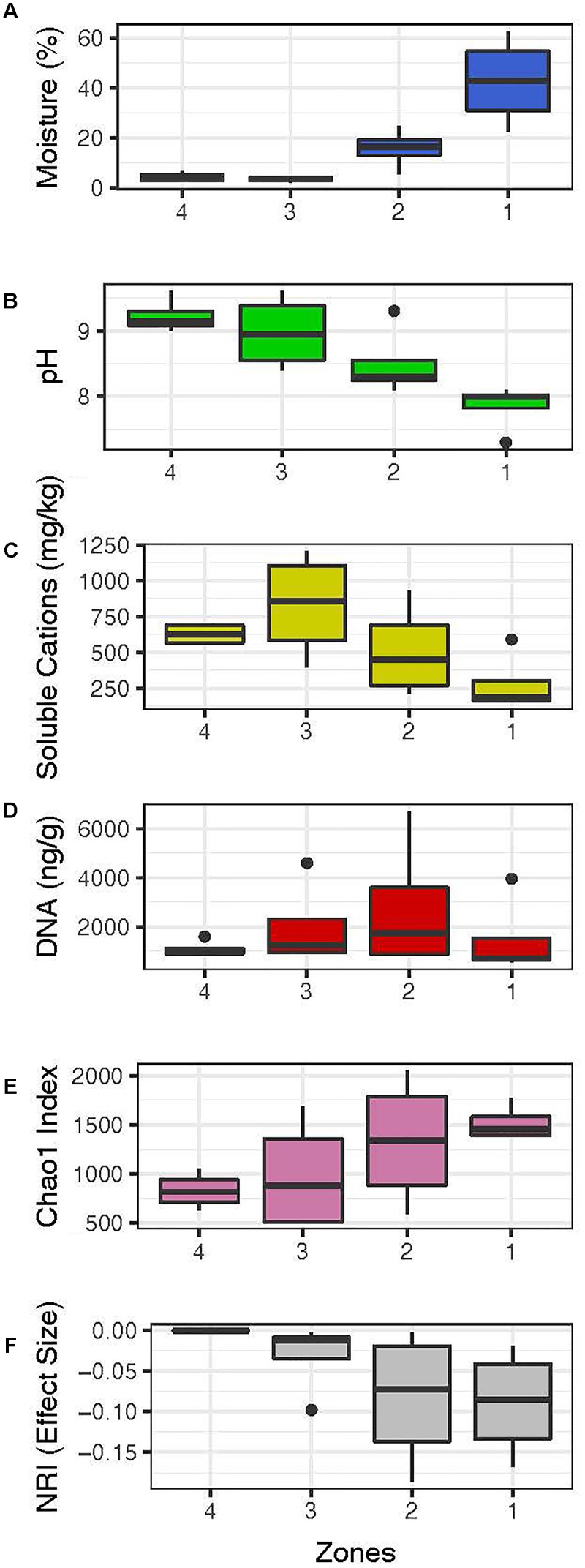
Summary of abiotic and biotic trends for soil moisture zones, from driest furthest from the pond (zone 4, 2.2%) to wettest at the water’s edge (zone 1, 62.56%). Full data for abiotic and biotic data at every moisture increment within each of the zones 1–4 are given in Figures 2, 6, 7.

Models for regression fitting estimated taxa (OTU) richness (Chao1 index) and diversity (Shannon’s index) against soil moisture(%) were compared and selected based on *p*-values, adjusted *R*^2^ values, and Akaike information criterion (AIC) of the models. In both cases, asymptotic regression model through the origin (SSasympOrig in R) was chosen. However, due to evidence of heteroscedastic residual variances with Shannon’s diversity data, its model was fitted with a variance power weighting function. The relationships between the relative abundances of major phyla (overall mean relative abundance > 2%, i.e., Acidobacteria, Actinobacteria, Bacteroidetes, Chloroflexi, Cyanobacteria, Firmicutes, and Proteobacteria) and moisture (%) were investigated using Spearman’s rank order correlation, due to violation of assumptions for Pearson’s correlation (homoscedasticity and linearity). Cyanobacteria and Actinobacteria showed significant correlation (*p* < 0.05) confirming monotonic relationships (positive and negative) between the phyla and moisture content in the soil. *PERMANOVA* was performed using the *adonis2* function in R “vegan” package (Oksanen et al., 2015) to analyze partitioning of community distance matrix among sources of variations. The analysis aims to test the significance of factors associated with samples (explanatory variables such as moisture and pH) in explaining the degrees of difference between communities. Terms including moisture, pH, and soluble cation (combining Mg, K, and Na measurements as a proxy of salinity) were added sequentially to the test. Phylogenetic trees for community phylogenetic structure analysis was constructed with FastTree v2.1.9 (Price et al., 2010). Representative OTU sequences were aligned by MUSCLE v3.8.31 (Edgar, 2004). Net relatedness index (NRI) was calculated based on mean phylogenetic distance (MPD) from the tree. Specifically, the MPD index quantifies mean distance of any taxon from every other taxon and was converted to NRI by multiplying with -1. Null model algorithm based on independent swap (999 randomization) was used to test whether communities were phylogenetically clustered (positive values) or overdispersed (negative values). Results for NRI (Figure 1F) was expressed as effects size, i.e., [–(MPD –MPDnull)/SD(MPDnull)]. In significantly clustered communities, OTUs are phylogenetically closer than expected under random sampling (i.e., MPD would be smaller than the average null MPD and thus NRI would be positive and larger than expected under the null model). Bacterial phylogenetic metrics were calculated using the R package “picante” (Kembel et al., 2010) and other package that support phylogenetically informed statistical analyses (“ape,” “phylobase,” “adephylo,” “phytools”) (Swenson, 2014). Detecting non-random phylogenetic patterns with metrics such as the Net Relatedness Index would reject the null hypothesis that bacterial taxa associate randomly in terms of phylogenetic relationships. Rejecting this null hypothesis would support, while not prove, the alternative hypothesis that non-random patterns can be generated by selection for shared traits (assuming trait conservatism). Selection can be exerted either by interactions between taxa, or the environment, or both. We decided that non-random phylogenetic patterns discriminated using Net Relatedness Index was a more robust method than network analysis where interactions are assumed or should be assumed *a-priori* or demonstrated experimentally before the analysis (Pascual and Dunne, 2006; Caruso et al., 2012) and would not allow us to prove biotic interactions while we would also lose the information embodied in the phylogeny of our taxa. In this case using Net relatedness Index could determine that phylogenetically related bacterial taxa share traits that are key to adapt to the extreme conditions of Antarctic soil. Thus, the fact that bacterial taxa do not associate randomly in terms of phylogenetic relationships imply potential selection for shared traits (either by interactions between taxa or the environment, or both). We believe there is more information in phylogeny based species distribution (community phylogenetics) than just species distribution (which would be the only trait considered by analyzing the species correlation matrix with network analysis).

## Results

### Clearly Defined Abiotic Gradients Occurred in the Hyporheic Zone

**FIGURE 2:**
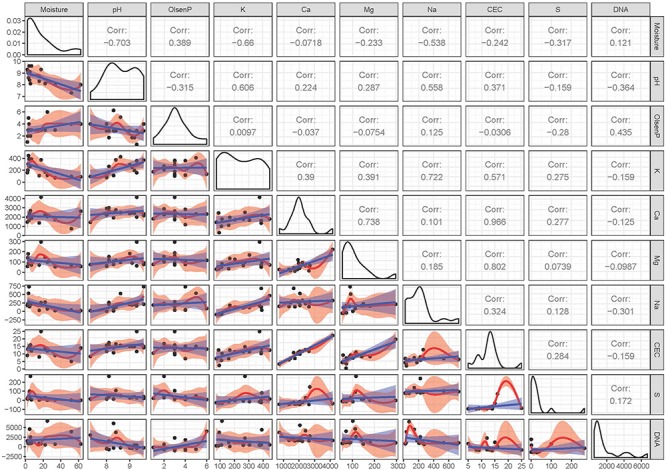
Pairwise comparison of multivariate geochemistry data from the soil samples. The upper diagonal displays the correlations, the diagonal boxes show the densities, and a matrix of scatterplots are shown in the lower diagonal. Linear regressions and 95% confidence intervals in the scatterplots are represented by blue lines/areas. Red lines/areas represent local regressions and their 95% confidence intervals. Moisture was measured in percentage; pH in pH unit; Olsen P (bicarbonate-extractable P), K, Ca, Mg, Na, and S (Sulfur and Sulfate) were measured in mg per kg of soil; cation-exchange capacity (CEC) was measured in me per 100 g of soil. DNA yield was measured in ng/g soil.

**FIGURE 3:**
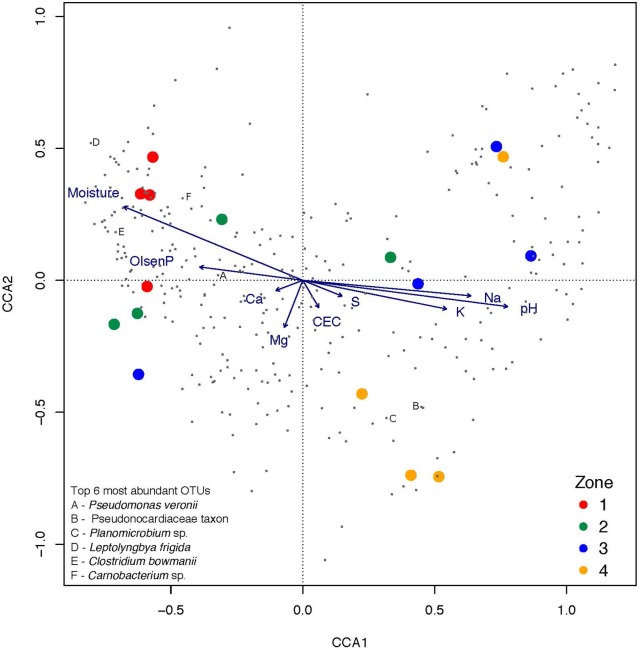
The relationship between samples, OTUs, and geochemical factors visualized via canonical correspondence analysis (CCA) with type III (symmetric) scaling. Samples from the zones, ranging from closest (1) to furthest (4) away from the pond are represented by color-coded circles and individual OTUs are represented as dots. Top six overall most relatively abundant OTUs are specifically marked. Quantitative explanatory variables are represented by as arrows. Proximity of the samples and OTUs to the direction of the arrow indicates likelihood the response variables are to be found with the geochemical factor.

The key abiotic driver of the hyporheic soil landscape was moisture [*PERMANOVA*, *R*^2^ = 0.31, *F*_(1,12)_ = 6.7603, *p* = 0.003; Table 1 and Figures 1–3] and moisture data supported the broad delineation of four zones in the hyporheic soil (Figure 1A). The gradient of moisture decreased sharply from over 60% at the water’s edge to approximately 2% in soils at the transition to arid soil. A steep transition occurred between 6.8 and 15.6% moisture content across all samples (*n* = 16). Other variables including pH (Figure 1B) and soluble cations, a proxy for osmotic challenge in soils, (Figures 1C, 2, 3) also varied along the transects but were not significant after accounting for moisture [*PERMANOVA p* = 0.062 [pH], *p* = 0.472 9 (soluble cations); Table 1]. These were identified as closely linked covariables with moisture and thus all subsequent analysis employed moisture as the primary abiotic variable. The low variability in other abiotic variables along transects did not display a significant trend.

**Table 1:** *PERMANOVA* test statistics of the main environmental factors influencing partitioning between communities.

	Degrees of freedom	Sum of squares	*R*^2^	F	Pr > F
Moisture	1	0.14843	0.30558	6.7603	0.003
pH	1	0.05698	0.11731	2.5953	0.062
Mg/K/Na	1	0.01684	0.03468	0.7672	0.472
Residual	12	0.26347	0.54243		
Total	15	0.48572	1.00000		

*adonis2 (formula, ps.d ∼ Moisture + pH + MgKNa; data, ps.df)*.

### Bacterial Diversity Displayed a Non-linear Response to Moisture

**FIGURE 4:**
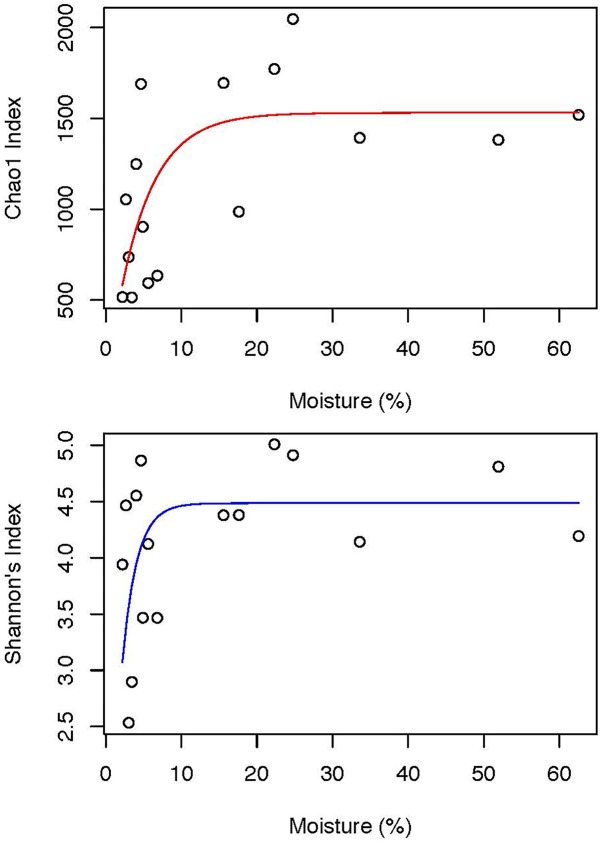
Asymptotic trends of Chao1 species richness estimator (Adj *R*^2^ = 0. 0.3875, *p* = 0.00278) and Shannon’s diversity index with soil moisture content (Adj *R*^2^ = 0.0679, *p* = 0.02748). For more detailed description of the models, please refer to Table 2.

Biomass estimation in poorly colonized desert soil is notoriously problematic and so we used recoverable environmental DNA as a proxy for biomass. These suggested relatively low biomass in all samples although those with intermediate sub-saturated moisture levels supported highest DNA yield (Figure 1D). Alpha diversity displayed a clear positive correlation with moisture-defined zones (Figure 1E) and analysis of bacterial phylogenetic structure revealed a trend toward random community assembly as moisture decreased within zones more distant from the water’s edge (Figure 1F). Deeper analysis using regression models revealed a striking non-linear response to moisture for alpha diversity (Figure 4 and Table 2). At low soil moisture levels of < 6.8%, Chao 1 richness estimates and Shannon’s diversity index increased sharply with small increments in soil moisture. At higher moisture levels of up to 62.6%, even large increases in moisture resulted in relatively little change in alpha diversity suggesting communities had reached an asymptotic state and were no longer limited by water.

**Table 2:** Asymptotic regression analyses of estimated species richness and diversity showing significant correlations.

	*R*^2^	Adjusted R^2^	AIC	logLik	L(χ^2^)	df	*p*-value
**Chao 1 species richness**					
nls(Chao1 ∼ SSasympOrig(moisture, Asym, lrc), data = alpha).					
Current model	0.4283	0.3875	240.00	-117.00	8.947	1	0.00278
Intercept model			246.95	-121.47			
**Shannon’s diversity index**					
gnls [Shannon ∼ SSasympOrig(moisture, Asym, lrc), data = alpha, control = gnlsControl(nlsTol = 0.05), weights = varPower(form = ∼ moisture)].					
Current model	0.13	0.0679	34.70	6.7603	-13.35	2	0.02748
Intercept model			37.89	2.5953	-16.94		

*AIC, Aikake information criterion; logLik, log likelihood; L(χ^*2*^), Chi-squared statistic*.

**FIGURE 5:**
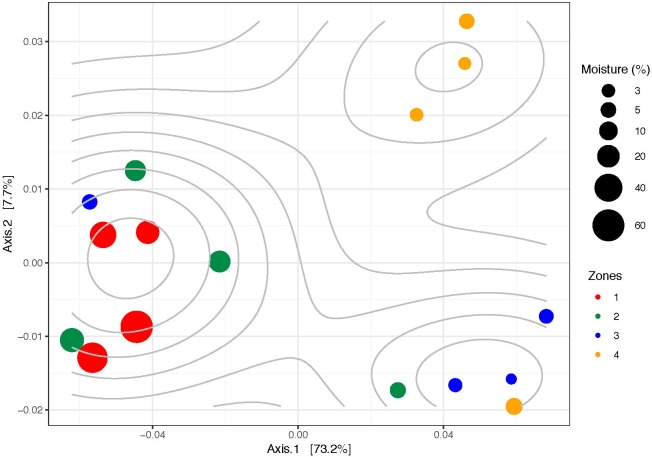
Community similarities visualized via principal coordinate analysis (PCoA) with weighted UniFrac distances. The size of each circle indicates the soil moisture (%). Contour lines were generated by 2D kernel density estimation and highlight the clustering of communities.

Community structure was clearly delineated by principal coordinates analysis (PCoA) and weighted UniFrac (biotic data) into three groupings that broadly reflected moisture-defined habitat zones within the hyporheic soils (Figure 5). The contrast between highest moisture soils cluster and lower moisture soil clusters (zones 1–2 vs. zone 3–4) explained 73% of overall community dissimilarity, whilst the other clusters for wet/ephemerally wet soils (zones 3–4) and dry soils (zone 4) explained less than 10% of community dissimilarity. Canonical correspondence analysis (CCA) was used to verify that the abiotic gradient due to moisture and its covariables was the most significant factor in community assembly and taxon occurrence (Figure 3).

### Response to Moisture Varied Markedly Between Taxonomic Groups

**FIGURE 6:**
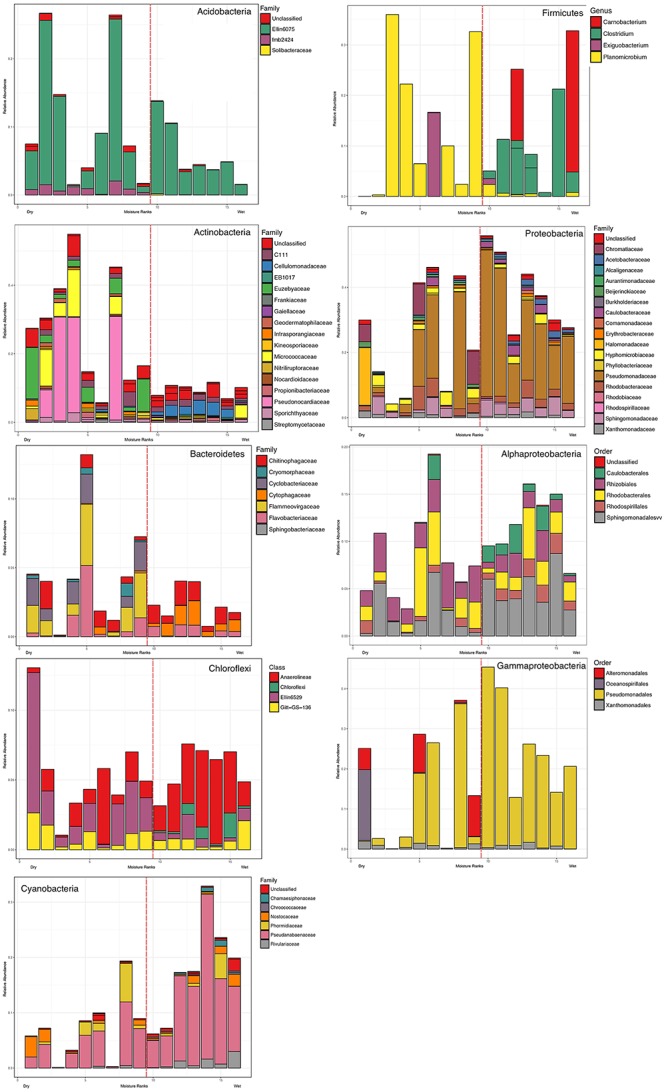
Taxa distribution of soil arranged in the order of the moisture gradient (lowest, 2.2% to highest, 62.56%). The stack bar plots show relative abundance of the taxa of interest to total bacterial abundance. Detailed taxonomic levels therein (e.g., Order, Family, Genus) are shown to illustrate response to moisture at lower taxonomic rank for abundant phyla. The dotted red line indicates the moisture threshold where shifts in member taxa were most common. The position of the dotted red line in each plot is based on rank order position of the threshold between sample ranked 9–10 in moisture content (dry to wet) which corresponds to the 6.82–15.57% threshold.

**FIGURE 7:**
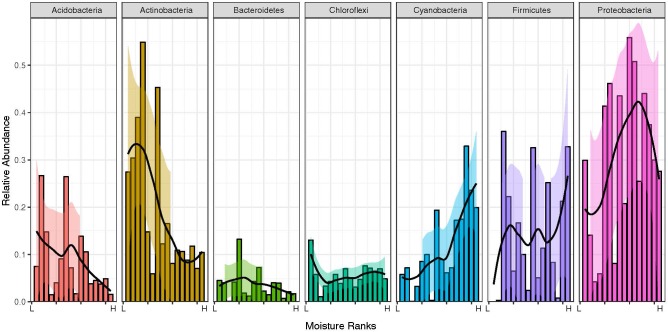
Visualization of changes to the relative abundance of major phyla (overall mean relative abundance > 2%, i.e., Acidobacteria, Actinobacteria, Bacteroidetes, Chloroflexi, Cyanobacteria, Firmicutes, and Proteobacteria) across the moisture gradient. The samples were arranged from driest (L, 2.2% moisture) to wettest (H, 62.6% moisture). The fit lines were generated via local regression and the shaded areas represent 95% confidence intervals. Taxonomic rank to Family, Order, Genus for taxa from individual samples is detailed in Figure 6.

We identified 19 bacterial phyla, and among these seven were above 2% mean relative abundance (Figure 6). These were considered as major phyla and subjected to further analysis. Five distinct patterns in response to the moisture gradient were identified (Figure 7). The Acidobacteria and Actinobacteria decreased in overall relative abundance as moisture increased, and displayed evidence for a succession of taxa within each phylum. Bacteroidetes and Chloroflexi displayed no discernible change with moisture and this in part reflected their low overall relative abundance. The Cyanobacteria increased in relative abundance with increasing moisture (Spearman’s correlation for the two major phyla: Cyanobacteria S = 174, p = 0.001, rho = 0.744; Actinobacteria S = 1134, *p* = 0.006, rho = -0.668) whilst maintaining overall taxonomic complexity. The relative abundance of Firmicutes was stochastic across the moisture range. Proteobacteria exhibited an increase in relative abundance with moisture, peaking at 15.57% moisture and decreasing at high levels. For most phyla an abrupt shift occurred between 6.82 and 15.57% moisture where the abundance of multiple taxa changed markedly (For example, within the Actinobacteria, Euzebyales were abundant only below 6.82%; Chloroflexi were abundant only above 15.57%, and among the Firmicutes, a transition in dominance occurred from *Planomicrobium* at lower moisture levels to *Clostridium* above 6.82% moisture.

## Discussion

### A Tipping Point for Bacterial Diversity at Low Moisture Levels

The steep response of diversity at low moisture levels indicated dynamic communities that were highly responsive to moisture. We identify 6.8% soil moisture as a tipping point below which extreme challenge to maintenance of diversity occurs. Previous studies in polar and non-polar deserts have reported differences between “wet” and “dry” soils without resolution of a contiguous moisture gradient, although comparisons with alpha diversity corroborate our findings (Pointing et al., 2007; Zeglin et al., 2011; Niederberger et al., 2015b). The tipping point occurred at a moisture level regarded as very low for edaphic systems globally (Pointing and Belnap, 2012) although it is typical for Dry Valleys soils and thus the response is relevant on a landscape scale to this Antarctic system.

The influence of pH and soluble salts has also been previously shown to be a major biological determinant across global datasets (Fierer and Jackson, 2006; Lozupone and Knight, 2007), although our study shows that at least for polar desert systems this likely reflects moisture availability since they strongly covaried. Our findings indicate that the fragile and endangered Antarctic terrestrial ecosystem (Cowan and Tow, 2004; Chown et al., 2012) is highly susceptible to shifts in biodiversity under low moisture regimes but that after a threshold soil moisture content of around 25% was reached then limited additional biological change would occur with increasing moisture. This highlights the immediacy of the threat to bacterial diversity from even small increases in liquid water availability due to climate change.

### Biotic Interactions Become Significant at Higher Moisture Levels

We observed phylogenetic patterns consistent with potential over-dispersion in wetter soil and high interdependence in taxon distribution (Kembel and Hubbell, 2006; Kembel, 2009; Cadotte et al., 2010). These patterns could be caused either by limited dispersal or distantly related taxa, or intense and possibly negative biotic interactions, or strong selection by environmental filtering, or a combination of both processes (Harper et al., 1961; Kembel, 2009). The scale of the study and the dispersal capability of Antarctic soil bacteria (Bottos et al., 2013) sugest that limited dispersal may play a minor role although we cannot rule this out completely. Instead, the wetter communities were dominated by Cyanobacteria and formed significantly non-random assemblages, where an increase in biocomplexity may also be accompanied by greater structural complexity that may influence community assembly (De Los Ríos et al., 2004, 2014) and be phylogenetically non-random due to the shared traits that are necessary to adapt to the extreme environment. This implies that biotic traits may be important in influencing the high degree of cyanobacterial endemism in Antarctic soils (Jungblut et al., 2010; Vyverman et al., 2010; Bahl et al., 2011). Other taxa likely to be influenced by biotic interactions include four out of six most abundant taxa in our study, all of which were psychrophilic species previously identified only from Antarctica (Franzmann et al., 1991; Spring et al., 2003; Reddy et al., 2004), which suggests competitive traits may be desirable in endemic taxa.

In contrast, stochastic process was more pronounced in drier soil i.e., the phylogenetic structure of taxa present was closer to being randomly assembled. Stochasticity may be particularly relevant to explaining occurrence of the spore-forming bacteria and other taxa tolerant of environmental stress via poikilohydric responses. For example, the Firmicutes exhibited a particularly high variance in relative abundance throughout the moisture range and particularly low diversity with only six taxa across two endospore-forming genera. We speculate that this accounted, at least in part, for the stochastic signal by reflecting recolonization from endospore reservoirs following dormancy events where low moisture resulted in extinction for other taxa.

### Implications for Ecosystem Functionality and Resilience

A fairly tight coupling between taxonomy and functionality has been shown for Antarctic Dry Valleys soil bacteria (Chan et al., 2013; Wei et al., 2016) as well as other systems (Delgado-Baquerizo et al., 2016; Fernández Martínez et al., 2017) and so the taxonomic shifts observed in this study may also reflect shifts in ability to conduct some geobiological transformations. The steep nature of the relationship between moisture and diversity below the tipping point suggests major changes are more likely at low moisture levels. The cyanobacteria-dominated communities supported by high moisture result in net carbon input to the system and this has a potential positive feedback on other trophic levels through provision and utilization of photosynthetic exudates (Niederberger et al., 2015a). This may facilitate persistence of cyanobacterial mats over multiple growing seasons despite relatively low productivity rates per unit biomass (Vincent and Howard-Williams, 1986; Conovitz et al., 1999). Rates of genetic substitution have been linked to water availability and productivity and therefore the diversity pattern we reveal might be due to an elevated tempo of evolution and speciation in wetter soils (Gillman and Wright, 2014). A shift toward more productive soils may lead to accelerated speciation over millennia. However, the more immediate impact would likely be a loss of polar desert-adapted endemicity that has evolved with low moisture or stochastic moisture stress. Given that recruitment estimates to Antarctic soils are extremely low (Burrows et al., 2009) this may reduce overall resilience to further shifts in moisture regime. A further concern is that since we have shown community shifts may occur in response to relatively small changes in moisture and at low moisture levels, the propensity for ‘greening’ of the Antarctic Dry Valleys in a warmer world should therefore be considered a risk in light of the nature of this system as a unique and protected hyper-arid environment (SCAR, 2004).

### Stochastic Patterns May Be Deterministic at Taxonomic Resolution Beneath Phylum Level

Our findings have broad implication for understanding the mechanistic basis for microbial community assembly. We highlight the critical importance of scale in taxonomic analysis and demonstrate it has fundamental impact on the outcome of estimates for the relative influence of stochastic and deterministic processes on community assembly. The widely accepted dogma is that microbial communities are largely driven by deterministic processes, although most microbial ecology studies have restricted their consideration of these to environmental filters (Zhou and Ning, 2017). Another important deterministic influence, however, is biotic interaction since deterministic drivers include all non-random processes. Our study showed that in a simple system moisture availability was the key abiotic process, but that this obscured the strong influence of biotic interactions at finer taxonomic resolution. This has largely been overlooked by the scale of taxonomic interrogation in many earlier studies of Antarctic microbial ecology, as most have focused on phylum or class level patterns. Stochastic processes also occupy a key role in our system (Volkov et al., 2003; Chave, 2004), especially for oligotrophic or resource-limited habitats where niche factors dominate but there also remains a relative large amount of random variation that is not a function of environmental variables. Some of this unexplained variance can be resolved when data at the phylum level are further decomposed into data at the family or genus level. For example in the case of Chloroflexi and Firmicutes in our model system their distribution appeared stochastic at phylum level but genus-level distribution was clearly a result of deterministic influence. Overall, we advocate broader consideration of deterministic and stochastic factors should be accompanied by fine-scale taxonomic resolution in order to yield a more comprehensive understanding of microbial interactions.

## Author Contributions

ML and SP conceived the study. CL, SC, and SP secured research funding. CL led the expedition team in the McMurdo Dry Valleys. LG and ML conducted the fieldwork. KL and SA performed the laboratory experiments. KL, SA, TC, and SP performed data analysis and interpretation. SP wrote the manuscript. All authors read and commented on the draft manuscript.

## Conflict of Interest Statement

The authors declare that the research was conducted in the absence of any commercial or financial relationships that could be construed as a potential conflict of interest.

## Footnotes

1 https://www.landcareresearch.co.nz/resources/laboratories/environmental-chemistry-laboratory/services/soil-testing/methods (accessed April 2018).
